# The Demands for Tooth Hygiene

**Published:** 1901-07

**Authors:** William H. Potter

**Affiliations:** Boston


### Reviews of Dental Literature.

"The Demands for Tooth Hygiene." By Dr. Ernst Jessen, Strassburg.[2]—The contents of this valuable article can best be given by a few quotations of important passages. In speaking of the advantages of public hygiene the author says, "The necessity of a systematic, well-regulated care of the health of the people has not as yet received general recognition. An intelligent care of the people's health must include a care of all conditions of life, as, for example, of dwelling-houses, of clothing, of food, of school hygiene. The care of the health of the people must begin with

---

[2] Zahnhygienische Forderungen, von Dr. med. Ernst Jessen, Deutsche Monatschrift für Zahnheilkunde, 6. Mai, 1901.

the children, in order to make their bodies strong and well developed, and to protect them against all sorts of pathogenic influences. For health is the marrow of the people. The interest in public hygiene has recently and in a marked way engaged the attention of the dental profession. The examinations conducted among school children and soldiers bear witness to this. These examinations have shown how necessary and important attention to dental hygiene is for the public health, and also show that the public school is the only proper place for such care. The improvement of the public health through better care of the teeth can only be accomplished in the school. For this reason the authorities must take the matter in hand. A regular yearly examination of the teeth will be sufficient. In some cities this is accomplished. In Strassburg all school children—sixteen thousand in number—have a yearly dental examination."

The author then proceeds to describe the method by which the results of these examinations are registered and the way in which necessary work on the teeth is performed at the clinic connected with the University of Strassburg. Each child is also furnished with a card containing twelve maxims in regard to the care of the teeth, and I append a translation of them, as follows:

1. At the age of two and a half years every child has twenty teeth.

2. At six years the large permanent grinding teeth appear in the back part of the mouth.

3. From the seventh to the fourteenth year the change lasts.

4. In the twelfth year appears the second large grinding tooth. Between eighteen and forty appear the wisdom-teeth.

5. Sound teeth are necessary for the stomach and the health of the entire body.

6. The milk-teeth have for the child the same value as the permanent have for the adult.

7. From youth up the teeth must be thoroughly brushed every morning and especially every evening.

8. Each year ought the teeth to be examined by a dentist.

9. As soon as there are cavities, they must be filled before pain appears.

10. Sound milk-teeth are necessary for sound permanent teeth.

11. In order to keep the oral cavity healthy, all roots which cannot be filled should be extracted.

12. The natural teeth must be preserved, since artificial teeth are only a help in necessity.

What has been accomplished for the care of the teeth of school children in the city of Strassburg should, according to the author, be accomplished in every German city. And he calls upon teachers to instruct their pupils in the use of the tooth-brush. And where poverty precludes the use of a brush, the practice of rinsing the mouth with a salt-and-water solution should be insisted upon.

Finally the author dwells upon the necessity of the care of the soldiers' teeth and of the care of the teeth of patients in hospitals.

WILLIAM H. POTTER, D.M.D.

BOSTON.

